# Circulating levels of miR125a, miR126, and miR146a-5p in patients with obstructive sleep apnea and their relation with markers of endothelial dysfunction

**DOI:** 10.1371/journal.pone.0287594

**Published:** 2023-11-02

**Authors:** Reza Fadaei, Soudabeh Fallah, Mohammad-Taher Moradi, Masoumeh Rostampour, Habibolah Khazaie

**Affiliations:** 1 Sleep Disorders Research Center, Kermanshah University of Medical Sciences, Kermanshah, Iran; 2 Department of Clinical Biochemistry, Faculty of Medicine, Iran University of Medical Sciences, Tehran, Iran; Lewis Katz School of Medicine, Temple University, UNITED STATES

## Abstract

**Background:**

obstructive sleep apnea (OSA) is a prevalent sleep disorder that is associated with increased risk factors for cardiovascular diseases (CVDs). Oxidative stress, insulin resistance, inflammation, and endothelial dysfunction are increased in OSA patients and microRNAs (miRs) are regulatory elements that influence these pathological mechanisms. miR125a, miR126, and miR146a-5p play a role in these pathological mechanisms and have not been evaluated in patients with OSA.

**Method:**

This case-control study was performed on 90 OSA patients and 34 controls. Circulating levels of miR125a, miR126, and miR146a-5 were determined using real-time PCR, and serum levels of hsCRP, ICAM-1, and VCAM-1 were evaluated using ELISA kits.

**Results:**

miR125a and miR146a were elevated in patients with OSA compared to controls while miR126 decreased significantly. All three miRs indicated a remarkable difference between the mild-OSA group compared to the severe-OSA group. Furthermore, patients with OSA showed elevated levels of hsCRP, ICAM-1, and VCAM-1. Multiple linear regression indicated an independent association of miR125a with ICAM-1 and hsCRP, miR126 associated with VCAM-1 and total cholesterol, and miR146a-5p represented an association with apnea-hypopnea index and ICAM-1. Furthermore, miR146a-5p illustrated a good diagnostic ability to differentiate between OSA and controls.

**Conclusions:**

Circulating miR125a, miR126, and miR146a-5p fluctuations in patients with OSA and their relations with markers of endothelial dysfunction provide in vivo evidence and suggest a potential role for these miRs with endothelial dysfunction in patients with OSA.

## Introduction

Obstructive sleep apnea (OSA) is one of the most prevalent sleep disorders which not only has an adverse impact on quality of life but also imposes several cardiovascular risk factors on the affected people [1]. Studies have reported a range of OSA prevalence from 9% to 38% and its prevalence increased with age, obesity, and being male [1, 2]. While the relationship of OSA with cardiovascular disease (CVD) is established the mechanism underlying this relationship is not fully understood. Several mechanisms have been proposed and investigated such as hypertension, endothelial dysfunction, insulin resistance, and dyslipidemia.

OSA is caused by recurrent partial or complete obstruction of the upper airway resulting in absence of inspiratory airflow [1]. This condition causes intermittent hypoxia which induces a situation like ischemia-reperfusion (I/R) results in increased ROS and oxidative stress [3]. Oxidative stress along with inflammation and sympathetic activation is considered the main driver of OSA consequences like insulin resistance, dyslipidemia, endothelial dysfunction, and atherosclerosis [1]. Exposing mice to intermittent hypoxia showed elevation of mitochondrial ROS that contributes to the development of type 2 diabetes mellitus [4]. In addition, intermittent hypoxia in rats leads to a decline in endothelial integrity and the number of endothelial cell progenitors [5]. Indeed, excessive ROS formation results in damage to biomolecules like DNA, proteins, and lipids in the body and in turn promotes an inflammatory cascade through transcription factor activation which causes upregulation of adhesion molecules and pro-inflammatory cytokines [6, 7]. Clinical studies have proven elevated inflammatory cytokines and adhesion molecules, which lead to endothelial dysfunction in OSA patients [8]. Moreover, the bioavailability of nitric oxide (NO) is reduced in intermittent hypoxia and in OSA patients which in turn promotes endothelial dysfunction [9].

Several mechanisms in the body regulate endothelial functions at transcription, post-transcription, and post-translation levels. MicroRNAs (miRs) are small, single-stranded, non-coding RNAs of 18 to 25 nucleotides that play a substantial role in the physiological and pathological process of the cells at the post-transcriptional level [10]. miRs can bind to 3’UTR of the target genes to induce their degradation and inhibit translation [10]. miRs were considered as a marker for response to therapy in OSA patients and a cluster of CVD-associated miRs called the HIPARCO-Score, comprising miR-378a-3p, miR-100-5p, and miR-486-5p showed a good ability to predict desirable response to CPAP treatment [11]. While there are studies that tested the ability of miRs to predict response to treatment, most studies evaluated the diagnostic potential of miRs. One of the first studies showed that miR-574-5p was upregulated, while 199-3p, miR-107, and miR-485-5p were suppressed, in patients with OSA in comparison to controls [12]. Moreover, miR-181a, miR-133a, miR-340, miR-199b, miR-486-3p, and miR-345 were found to be lower in the plasma of male OSA patients compared to controls [13]. In addition to the diagnostic ability of miRs in sleep apnea, it has been shown that miRs can affect pathophysiological pathways and mechanisms related to OSA. Circulating exosomes containing miRs from subjects who were exposed to intermittent hypoxia considerably upregulated ICAM-1 and downregulated endothelial nitric oxide synthase (eNOS) [14]. There are miRs that have close relationships with endothelial dysfunction, inflammation, and cardiovascular diseases which have not been evaluated in patients with OSA.

There is considerable literature that reported the relation of miR125a with underlying mechanisms of CVD [15], and lower levels of this miR have been reported in subjects suffering from insufficient sleep [16]. miR125a has been reported to have protective effects against I/R injuries in rats’ myocardium [17], moreover, miR125a has a protective role in the inflammatory process through the PYD domains-containing protein 3 (NLRP3) [18]. miR126 plays a role in endothelial proliferation and in developmental angiogenesis [19, 20], and reports indicate that miR126 is released by endothelial cells [21]. miR126 was found to be downregulated in senescent endothelial cells and inhibition of miR126 resulted in a decrease in HIF-1α protein levels, that disrupt the wound healing process [22]. Additionally, miR126 showed an inverse relationship with the VCAM-1 and was found to be downregulated in senescent human aortic endothelial cells [23]. Furthermore, miR126 was decreased in OSA patients with hypertension compared to OSA patients with normal blood pressure [24]; chronic intermittent hypoxia in rats results in a decrease of miR126a-3p and an increase in HIF-1α in the rat [25]. miR146a-5p is a key regulator of several cancers, including prostate, breast, and gastric cancer [26–28], in addition, the levels of miR146a-5p have been found to be higher in animal and cell models of I/R [29]. Furthermore, miR146a-5p exacerbates injury induced by IH in H9c2 cells by reducing cell viability and by increasing apoptosis through the X-linked inhibitor of apoptosis protein (XIAP) [30]. Furthermore, miR146a-5p suppressed endothelial activation and pro-inflammatory signaling in endothelial cells [31].

While there are shreds of evidence for the relation between these three miRs and endothelial dysfunction, there is no study on the association of these miRs with OSA. Therefore, the present study sought to measure circulating levels of miR125a, miR126, and miR146a-5p in patients with OSA to determine if they were related to markers of endothelial dysfunction and inflammation.

## Method

### Study population and diagnosis of OSA

This case-control study was performed on 124 subjects (90 OSA and 34 control) who underwent polysomnography (PSG) in the sleep clinic of Farabi Hospital in Kermanshah, Iran, from March 2020 until March 2022. The diagnosis was based on the results of PSG [32, 33], subjects with an apnea-hypopnea index (AHI) ≥ 5 were categorized as OSA patients. Controls were subjects with AHI < 5 and did not have any sleep disorders. Briefly, continuous PSG was performed overnight (7hrs) for all subjects via SOMNOscreen™ plus (SOMNOmedics GmbH, Randersacker, Germany). American Academy of Sleep Medicine (AASM 2012) guidelines were used to define hypopnea and apnea. Hypopnea is defined as reduced airflow by ≥30% along with reduced oxygen desaturation index by ≥3% or arousal and apnea are classified as a whole cessation of airflow for ≥10 seconds. The mean number of hypopneas plus apneas is considered AHI. The severity of the disease was defined according to the AHI value: 1) Mild: 5≤AHI<15 (n = 30), 2) Moderate: 15≤AHI<30 (n = 29), and 3) Severe: AHI≥30 (n = 30). Subjects with evidence or history of cardiovascular diseases, autoimmune diseases, cancer, and diabetes (according to the criteria of American diabetes association) were excluded from the study.

### Anthropometric and laboratory parameters

Fasting venous blood sample (5 mL) was obtained from participants, and serum was separated immediately by centrifugation and stored at—70° C. Systolic blood pressure (SBP) and diastolic blood pressure (DBP) were measured in a was seated position using a standard sphygmomanometer. Body mass index (BMI) calculated using standard formula: weight (kg)/height (m^2^). Levels of fasting blood glucose (FBG) and lipid profile, including triglyceride (TG), total cholesterol, low-density lipoprotein cholesterol (LDL-C), and high-density lipoprotein cholesterol (HDL-C) were determined using a spectrophotometric assay with commercially available kits (ParsAzmon, Tehran, Iran), and an auto-analyzer. Fasting insulin levels were measured using an enzyme-linked immunosorbent assay (ELISA) kit (Monobind, USA) according to the manufacturer’s instructions.

### Serum levels of adhesion molecules

ELISA kits (Quantikine, R&D Systems; USA) were used to measure serum levels of ICAM-1 and VCAM-1. Intra-assay and inter-assay coefficients of variation (CV) for ICAM-1 and VCAM-1 were <6.5% and <7% respectively. Moreover, the minimum detectable range for ICAM-1 and VCAM-1 were 0.096 ng/mL and 0.6 ng/mL, respectively.

### Determining miRs circulating levels

The miRNA was extracted from serum samples using the QIAzol reagent (Qiagen, USA) according to the manufacturer’s protocol. The concentration and purity of RNA were tested by a NanoDrop (Thermo Fisher Scientific, USA). The miR complementary DNA (cDNA) was synthesized using TaqMan Advanced miRNA cDNA Synthesis Kit (Applied Biosystems, USA). The levels of circulating miRs were measured using TaqMan Advanced miRNA Assays (Thermo Scientific, USA) based on the manufacturer’s protocol. miR-361-5p was used as the internal control and specific primers and probes were applied for each of the miRNAs. Relative quantification of miRs was determined by the 2^- ΔCt^ method [34].

### Statistical analysis

Statistical Package for the Social Sciences (SPSS) version 25 was used for statistical analysis. The chi-square test was used to compare categorical data between the groups and results are represented by frequency and percentage. The mean between two groups was compared using either Student’s t-test or Mann-Whitney U test, depending on the normality test results. One-Way ANOVA or Kruskal Wallis test were used to compare continuous variables between more than two groups according to normality results. Analysis of Covariance (ANCOVA) was performed to adjust for the possible impact of covariates on miRNA levels. Data that were not normally distributed were transformed logarithmically before including in correlation tests or regression analyses. The Pearson correlation test was used to test the correlation between miRs and other variables. The correlation for PLMS was tested using the Spearman test because there was a zero in PLMS. To identify the independent association between miRs and continuous variables, all variables that were found to be correlated were included in a linear regression analysis. Binary logistic regression was used to test the relation of miRs with the risk of OSA. The receiver operating characteristic (ROC) curve was plotted to test the diagnostic ability of circulating miRs. The sample size was calculated for a case-control study comparing the levels of three circulating miRs (miR146a, 126, and 125) between the OSA group and the control group separately. The calculation was performed with a power of 80% and a significance level (alpha) of 0.05, and the highest sample size required was determined. A p-value of less than 0.05 was considered statistical significance.

## Results

### The basic characteristic of the study population

Table 1 represents the detailed characteristic of the studied population. Age and BMI indicated no considerable difference between the groups. While patients with OSA had a bigger neck circumference (p = 0.019), waist circumference didn’t reach the significant threshold (p = 0.078). SBP and DBP were remarkably higher in the OSA group compared with controls (p<0.01 for both). As expected, both AHI and RDI were considerably higher in OSA patients compared to controls (p<0.001 for both). Moreover, total sleep time and sleep efficiency indicated no considerable difference between the groups while periodic limb movements in patients with OSA was higher compared to controls. While average SpO2 indicated no remarkable difference between the groups (p = 0.121), minimal SpO2 declined in patients compared to controls (p<0.001). FBG illustrated no considerable difference between the groups but, insulin and HOMA-IR showed a considerable elevation in the OSA group compared to the control group. TC and LDL-C were not significantly different between the groups. HDL-C was reduced in the OSA group compared to controls (p = 0.037) while TG was elevated in the patients compared to controls (p = 0.043).

**Table 1:** The basic characteristic of the studied population.

Variables	Control (n = 34)	OSA (n = 90)	P Value
Age (year)	44.56 ± 9.34	45.28 ± 12.85	0.733
Sex (male /female)	18 (52.1%)/16(47.9%)	63 (0%)7/27(30%)	0.06
BMI (kg/m^2^)	26.53 ± 3.25	26.93 ± 2.61	0.477
Neck circumference (cm)	37 (33, 39)	39 (37, 41)	0.019
Waist circumference (cm)	93.38 ± 9.37	96.86 ± 9.86	0.078
SBP (mmHg)	110 (110, 120)	120 (115, 125)	0.003
DBP (mmHg)	70 (70, 80)	80 (70, 80)	0.004
AHI (event/h)	2.2 (1.25, 2.9)	18.9 (9.25, 37)	<0.001
RDI (event/h)	4.82 ± 3.92	28.42 ± 20.53	<0.001
AverageSpO2 (%)	92.15 ± 4.57	89.79 ± 8.34	0.121
MinimalSpO2 (%)	89.26 ± 5.65	84.22 ± 9.33	<0.001
TST (h)	6.5 (6.29, 7.27)	6.54 (5.53, 7.33)	0.831
SE (%)	86.6 (81.1, 93.3)	86.45 (74.6, 93.7)	0.827
PLMS	0.9 (0, 3.2)	3.35 (0.7, 8.5)	0.001
Insulin (μU/ml)	3.2 (2.5, 4.4)	4.5 (2.45, 7.16)	0.009
HOMA-IR	0.73 (0.57, 1.09)	1.08 (0.57, 1.59)	0.010
FBS (mg/dL)	94.63 ± 11.428	95.84 ± 10.68	0.583
TC (mg/dL)	164.31 ± 34.37	167.03 ± 45.96	0.755
LDL-C (mg/dL)	94.82 ± 23.72	99.44 ± 31.69	0.383
TG (mg/dL)	121 (94, 157)	147 (109.5, 183)	0.043
HDL-C (mg/dL)	44.1 (41.5, 51.75)	42 (37, 48)	0.037

BMI, body mass index; SBP, systolic blood pressure; DBP, diastolic blood pressure; AHI, apnea-hypopnea index; RDI, respiratory disturbance index; averageSpO2, average saturation of peripheral oxygen; MinimalSpO2, minimal saturation of peripheral oxygen; TST, total sleep time; SE, Sleep efficiency; PLMS, Periodic limb movements in sleep; HOMA-IR, homeostatic model assessment for insulin resistance; FBS, fasting blood sugar; TC, total cholesterol; LDL-C, low-density lipoprotein cholesterol; TG, triglyceride; HDL-C, high-density lipoprotein cholesterol

### Circulating hsCRP and soluble adhesion molecules

**Fig 1:**
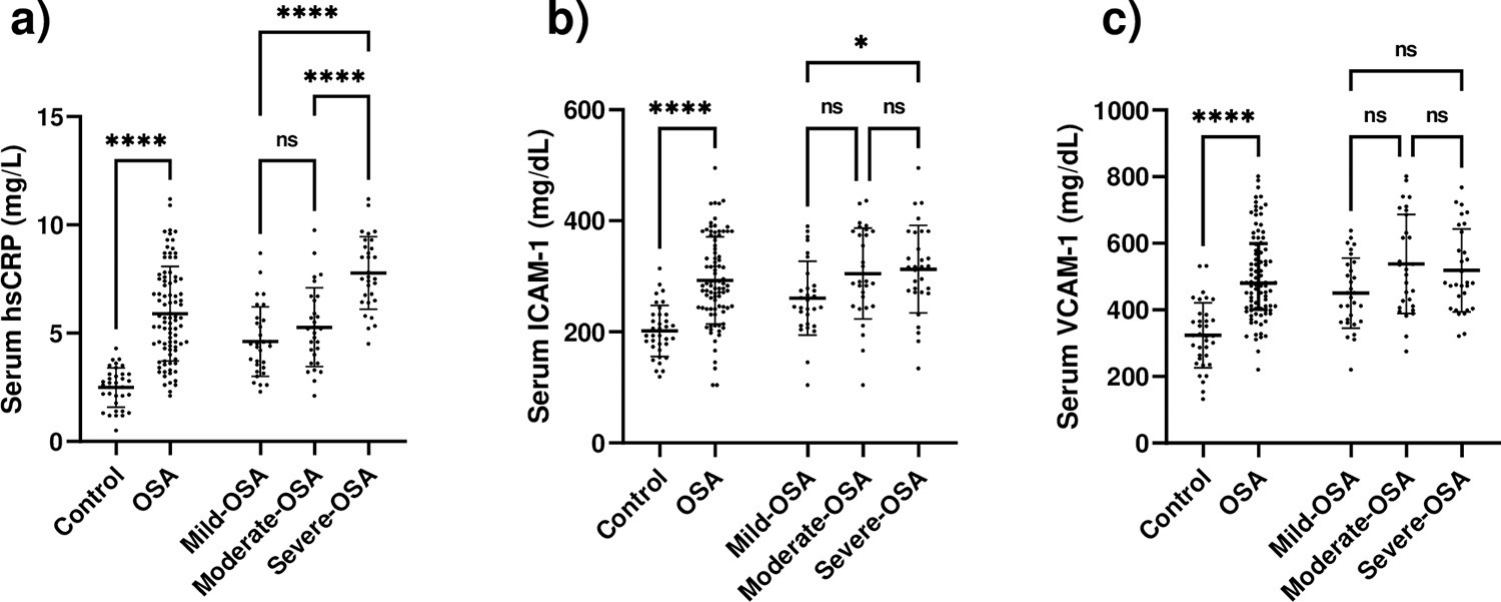
Serum levels of hsCRP, ICAM-1, and VCAM-1. a) Serum levels of hsCRP were elevated in OSA patients compared to controls, and the severe-OSA group represents a higher concentration of hsCRP compared with mild-OSA and moderate-OSA groups. b) Serum concentration of ICAM-1 was higher in the OSA group compared to controls, and the severe-OSA group indicated higher ICAM-1 compared to the mild-OSA group. c) Patients with OSA were found to have a higher concentration of VCAM-1 compared to controls. ns, not significant; * p<0.05, ** p<0.01, *** p<0.001 and **** p<0.0001.

Serum levels of hsCRP, as an inflammatory marker, were elevated in patients with OSA (5.86 ± 2.2) compared with controls (2.5 ± 0.92 mg/L, p<0.001). According to the comparison between OSA categories, severe OSA (7.78 ± 1.68 mg/L) had higher hsCRP levels than mild (4.62 ± 1.61 mg/L) or moderate OSA (5.27 ± 1.82 mg/L) (p<0.001 for both), but mild and moderate OSA did not differ significantly from each other (Fig 1A). ICAM-1 concentration was higher in OSA patients (200.23 ± 45.99 mg/dL) compared to controls (292.27 ± 78.12 mg/dL, p<0.001), furthermore, severe-OSA group indicated a higher concentration of ICAM-1 compared to mild-OSA (260.8 ± 66.30 mg/dL) (p = 0.048), and there was no considerable difference between moderate-OSA (304.9 ± 81.7 mg/dL) with mild-OSA and severe-OSA (312.9 ± 78.83 mg/dL) (Fig 1B). Similarly, VCAM-1 (480 (399, 599) vs. 322 (258.5, 387)) increased in patients with OSA compared to controls (p<0.001 for both), but there was no remarkable difference between mild-OSA (444 (364.5, 538)), moderate-OSA (521 (408.5, 692)) and severe-OSA (480.5 (404, 639.3)) groups (Fig 1C).

### Circulating MicroRNAs

**Fig 2:**
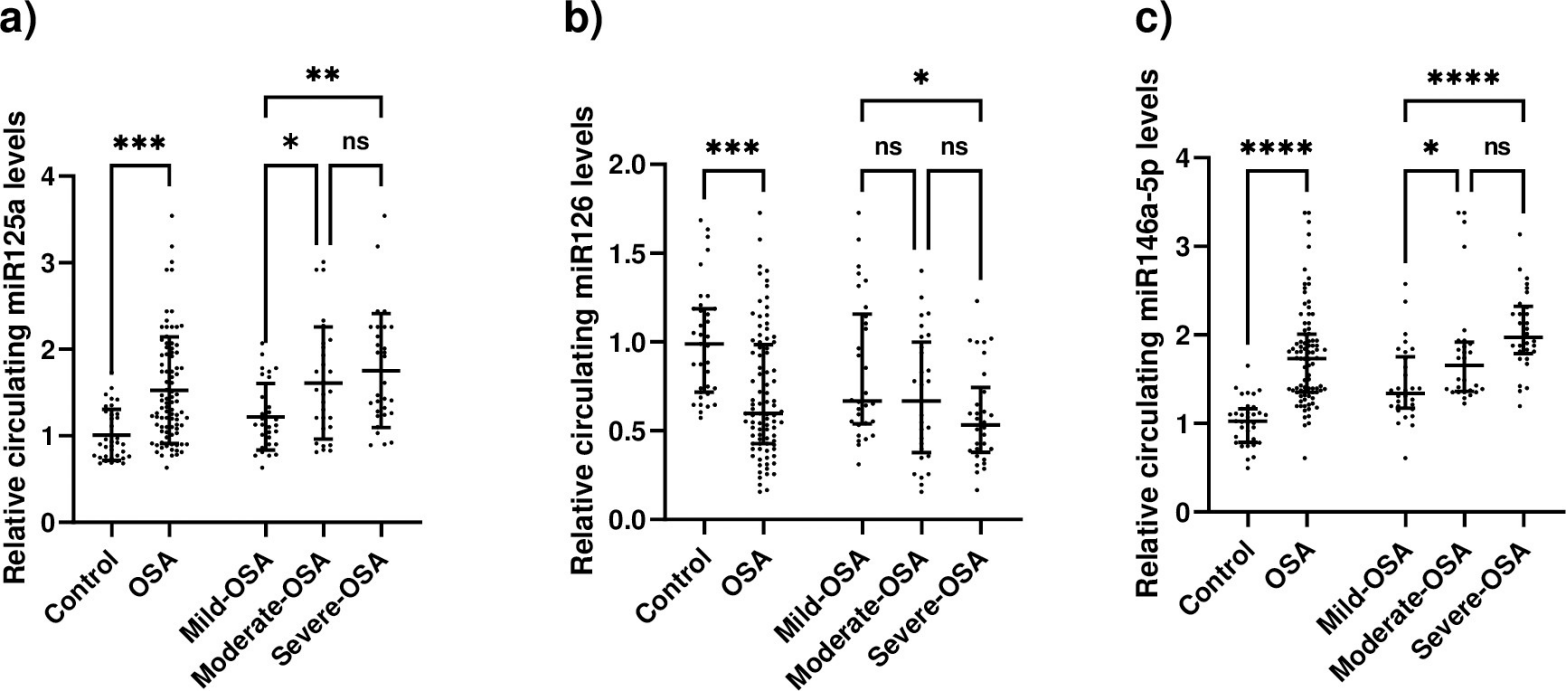
Circulating levels of miRs. a) miR125a levels increased in patients with OSA compared to controls, and in the severe-OSA group compared to the mild-OSA group. b) miR126 concentration was lower in OSA compared to controls and in the severe-OSA group compared to the mild-OSA group. c) circulating mi146a were found to be higher in OSA patients in comparison to controls, and mild-OSA had a lower concentration of miR146a-5p compared with moderate-OSA and severe-OSA groups. ns, not significant; * p<0.05, ** p<0.01, *** p<0.001 and **** p<0.0001.

The levels of circulating miR125a were higher in OSA patients than in controls (p<0.001). Moreover, patients with mild OSA had higher levels of miR125a than those in the moderate and severe OSA groups (Fig 2A). Conversely, the levels of miR126 were lower in patients with OSA than in controls (p<0.001), and miR126 was also decreased in the severe OSA group compared to the mild OSA group (Fig 2B). In addition, miR146a-5p levels were higher in OSA patients than in controls (p<0.001), but miR146a-5p was found to be lower in the mild OSA group compared to the moderate and severe OSA groups (Fig 2C). All the results remained consistent even after adjusting for age, sex, and BMI.

### Association of miRs with other variables

Correlation analysis was performed in each group separately and the results are shown in Table 2. In the patients with OSA, miR125a indicated a positive association with miR146a-5p, AHI, RDI, FBS, hsCRP, ICAM-1, and VCAM-1, and an inverse correlation with minimal SpO2. Furthermore, miR126 indicated an inverse correlation with AHI, insulin, HOMA-IR, TC, ICAM-1, and VCAM-1. miR146a-5p represented a positive correlation with AHI, RDI, neck circumference, FBS, insulin, HOMA-IR, ICAM-1, and VCAM-1, and an inverse correlation with minimal SpO2 and HDL-C. Multiple stepwise linear regressions were performed to find independent associations of miRs with correlated variables. miR125a was found to independently associated ICAM-1 [B (95% CI) = 0.002 (0.001, 0.004), p = 0.003] with hsCRP [B (95% CI) = 0.099 (0.047, 0.151), p<0.001] and miR126 showed an independent association with VCAM-1 [B (95% CI) = -0.651 (-1.050, 0.252), p = 0.002] and TC [B (95% CI) = -0.001 (-0.002, 0.000), p = 0.027]. Furthermore, miR146a-5p demonstrated an independent association with the AHI [B (95% CI) = 0.141 (0.067, 0.216), p<0.001] and ICAM-1 [B (95% CI) = 0.001 (0.000, .001), p = 0.007]. The relation of miRs with sex was tested and there was no considerable difference between men and women in terms of miR125a and miR126, however, miR146a-5p indicated a higher level in men compared to women.

**Table 2:** Pearson correlation of miR125a, miR126, and miR146a-5p with other variables.

Variables	Groups					
	Control			OSA		
	miR125a	miR126^#^	miR146a-5p^#^	miR125a	miR126^#^	miR146a-5p^#^
miR125a	1	-0.135	-0.183	1	-0.106	0.325**
miR126^#^	0.089	1	-0.059	-0.111	1	0.007
miR146a-5p^#^	-0.107	-0.059	1	0.333**	0.007	1
Age	-0.006	-0.122	0.297	0.091	0.055	0.152
BMI	0.078	-0.311	0.123	0.099	-0.133	0.154
AHI^#^	-0.263	-0.069	0.219	0.356**	-0.259*	0.428**
RDI	0.061	-0.242	0.164	0.352**	-0.176	0.435**
AverageSpO2	-0.044	0.004	-0.249	-0.026	0.100	-0.019
MinimalSpO2	-0.305	0.152	-0.091	-0.228*	0.000	-0.234*
TST^#^	0.257	-0.107	0.093	0.019	0.029	-0.063
SE^#^	0.260	-0.098	0.056	0.011	0.023	-0.060
PLMS^$^	0.116	-0.073	0.297	-0.147	-0.028	-0.041
Neck circumference	-0.057	-0.249	0.207	0.024	-0.110	0.216*
Waist circumference	0.110	-0.250	0.264	-0.028	-0.159	0.164
SBP^#^	0.167	-0.266	0.219	0.151	-0.045	0.038
DBP^#^	0.181	-0.336	0.142	-0.038	-0.129	-0.152
FBS	0.210	-0.118	0.185	0.220*	-0.076	0.219*
Insulin^#^	0.206	0.202	0.125	0.134	-0.267*	0.242*
HOMAIR^#^	0.238	0.168	0.158	0.162	-0.267*	0.265*
TC	-0.207	0.037	0.242	0.089	-0.291**	0.145
TG^#^	-0.063	0.172	0.194	0.035	-0.150	0.119
LDL	0.007	0.271	0.261	0.118	-0.120	0.198
HDL^#^	-0.226	0.080	-0.213	-0.164	-0.105	-0.213*
HsCRP	0.313	-0.132	0.288	0.384**	-0.073	0.189
ICAM-1	0.044	-0.064	-0.173	0.322**	-0.317**	0.357**
VCAM-1^#^	0.001	-0.100	-0.196	0.244*	-0.368**	0.266*

BMI, body mass index; SBP, systolic blood pressure; DBP, diastolic blood pressure; AHI, apnea-hypopnea index; RDI, respiratory disturbance index; averageSpO2, average saturation of peripheral oxygen; MinimalSpO2, minimal saturation of peripheral oxygen; TST, total sleep time; SE, Sleep efficiency; PLMS, Periodic limb movements in sleep; HOMA-IR, homeostatic model assessment for insulin resistance; FBS, fasting blood sugar; TC, total cholesterol; LDL-C, low-density lipoprotein cholesterol; TG, triglyceride; HDL-C, high-density lipoprotein cholesterol

* p<0.05

** p<0.01

# Logarithmically transformed

$ Spearman correlation test

### Association of miRs with OSA

The association of circulating miRs with the risk of OSA was tested using binary logistic regression. The results showed that levels of miR are associated with the risk of OSA and the relation remained significant after adjusting for age, sex, and BMI (Table 3).

**Table 3:** Odds ratio (OR) of the OSA presence according to circulating levels of miRs.

	Model	B	S.E.	Wald	OR	95% C.I.for OR		p
						Lower	Upper	
miR125a	Crude	2.874	0.713	16.245	17.705	4.377	71.616	<0.001
	Adjusted*	2.872	0.721	15.875	2.872	4.302	72.537	<0.001
miR126^#^	Crude	-2.227	0.606	13.486	0.108	0.033	0.354	<0.001
	Adjusted*	-2.275	0.624	13.293	0.103	0.030	0.349	<0.001
miR146a-5p^#^	Crude	7.324	1.556	22.166	1516	71.9	31990	<0.001
	Adjusted*	8.769	1.949	20.249	6430	141.092	293042	<0.001

# Logarithmically transformed

*An adjustment was performed for age, sex, and BMI.

**Fig 3:**
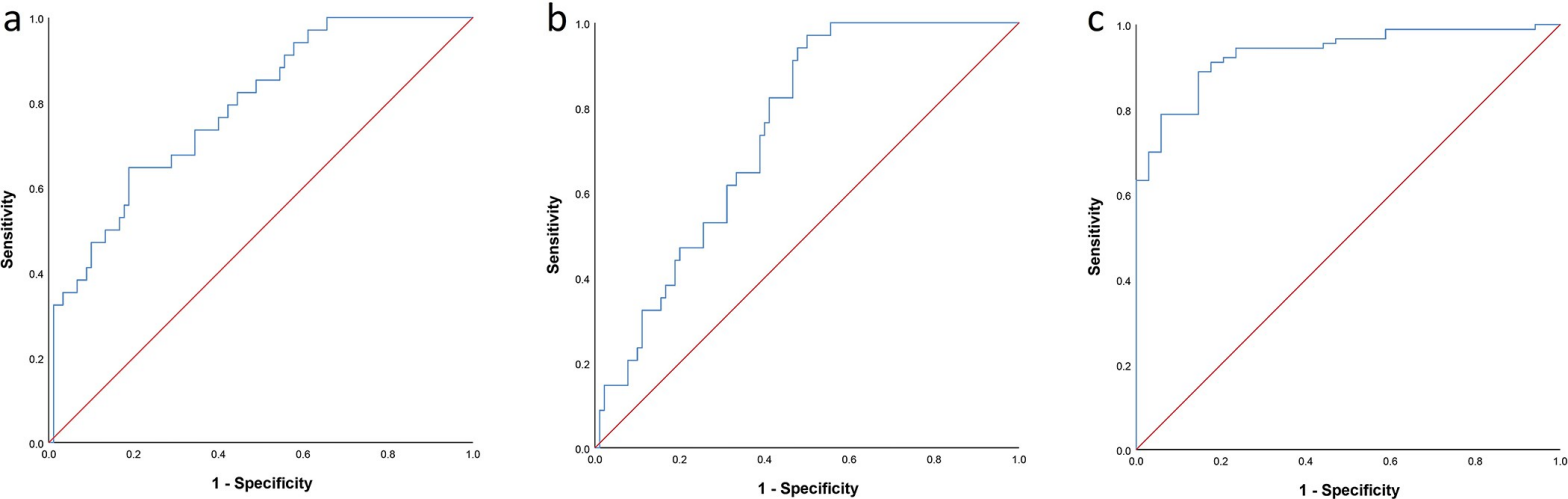
ROC curve for the diagnostic ability of a) miR125a, b) miR126, and c) miR146a-5p.

The diagnostic ability of the miRs was assessed using ROC analysis and the results showed that miR125a had a relatively good ability to distinguish between OSA and control [area under the curve (AUC) and 95% CI: 0.787 (0.702, 0.873), p<0.001] (Fig 3A). Similarly, miR126 represented a relatively good potential diagnosis of OSA [AUC and 95% CI: 0.745 (0.66, 0.83), p<0.001] (Fig 3B). Moreover, miR146a-5p had a high potential for OSA diagnosis with an AUC and 95% CI: 0.933 (0.890, 0.976), p<0.001 (Fig 3C).

## Discussion

The present study established a substantial difference in circulating levels of three miRs which are involved in vascular inflammation and endothelial cell dysfunction [35–37]. miR125a indicated a higher concentration in OSA patients and showed a positive association with disease severity. This is the first study of miR125a in patients with OSA while there are studies that have reported perturbation in circulating levels of miR125a in diseases conditions such as cancer and cardiovascular diseases [15, 38]. It has been established that the lack of this miR can lead to defects in the development of the cardiovascular system. In contrast with the present study, a decline in levels of miR125a was detected in systemic lupus erythematosus and juvenile-onset lupus patients [39]. Additionally, Hijmans et al, indicated a lower concentration of this miR125a in a subject suffering from insufficient sleep. It is worth to noting that there is no consensus on the circulating level of miR125a in different diseases, so it seems likely that miR125a has a complex regulation mechanism and disease setting can have a huge impact on circulating levels of miR125a. Interestingly, there was a significant positive correlation between miR125a and two important OSA-related parameters (i.e. AHI and RDI), with an inverse correlation with minimal SpO2. Repetitive hypoxia/reoxygenation is an important aspect of OSA pathogenesis, creating a condition similar to ischemia/reperfusion (I/R). There is evidence suggesting a role for miR125a in I/R [40]. Furthermore, miR125a has been shown to protect rat myocardium against I/R injuries through urocortin [40] and increased levels of miR125a may represent a response to combat such injuries [17]. On the other hand, miR125a modulates inflammation through the inhibition of tet methylcytosine dioxygenase 2 (TET2). This function of miR125a results in mitochondrial dysfunction, elevation of oxidative stress, activation of nuclear factor-κB, and increased generation of pro-inflammatory cytokines [41]. The results of the present study showed a positive relation between miR125a and hsCRP as a marker of inflammation. On the other hand, overexpression of miR125a resulted in a decline in ICAM-1 and VCAM-1 expression in human brain microvessel endothelial cells [42], and our result showed that miR125a positively correlated with markers of endothelial dysfunction (i.e. ICAM-1 and VCAM-1). This controversy may be due to the fact that increased levels of miR125a in vivo may not be as high as the levels achieved through *in vitro* overexpression, which could impact the observed effects of miR125a. There is inconsistency in how miR125a is related to inflammation, I/R, and markers on vascular function [43, 44]. While there is evidence for the protective role of miR125a against I/R injuries, and its ability to improve vascular function by reducing ICAM-1 and VCAM-1, it also has a negative impact on inflammation and oxidative stress [17, 43, 44]. Moreover, miR-125a inhibits Hyaluronan Synthase 1 [45], and clinical reports have shown that levels of hyaluronic acid reduced in patients with OSA, which is associated with inflammation and endothelial dysfunction in these patients [46]. Regarding these findings, our results suggest a compensatory increase in miR125a in response to I/R and endothelial dysfunction, but there is no evidence of the effectiveness of elevated miR125a to reduce levels of ICAM-1 and VCAM-1, and instead of miR-125a being a modulator of inflammation, it might be that inflammation upregulates miR-125a. These results suggest that the role of miR-125a in OSA patients and pathological conditions such as endothelial dysfunction is complex and requires further investigation.

miR126 represented a lower concentration in OSA patients compared to controls. This study presents the first report of miR126 in OSA patients. Our results showed a correlation between miR126 with AHI, which is the main indicator of OSA and its severity. Hypoxia was found to be a factor that downregulates miR126 in RF/6A cell line [47]. Previous studies have reported a lower concentration of miR126 in patients with type 2 diabetes mellitus and prediabetes [48]. Consistent with these findings, our results showed an inverse correlation between reduced miR126 and HOMA-IR and insulin levels in OSA patients. Moreover, miR126 is inversely correlated with markers of endothelial function in OSA patients (e. i. ICAM-1 and VCAM-1). Several lines of evidence have shown the beneficial impact of miR126 on endothelial function [49] which may explain the association between miR126 and ICAM-1 and VCAM-1 in the present study. A study found that miR126 downregulation leads to an increase in VCAM-1 expression in endothelial cells [49]. Another study reported that miR126 was lower in patients with intracerebral hemorrhage and that using miR126 mimics to downregulate VCAM-1 in the rat model of intracerebral hemorrhage [50]. In addition, miR126 increases endothelial cell viability and promotes activation of endothelial nitric oxide synthase (eNOS) by suppressing phosphoinositide 3-kinase (PI3K)/AKT/eNOS [51]. Collectively, miR126 reduction in patients with OSA and its relation with vascular adhesion molecules provide *in vivo* evidence for the association of this miR with the pathological aspects of OSA.

miR146a-5p was found to be present at higher levels in patients with OSA which demonstrated good diagnostic ability. Studies have reported that miR146a can mediate several endothelial pathophysiological mechanisms. miR146a was found to be upregulated under intermittent hypoxia and mediates its effects in H9c2 cells [30]. Similarly, miR146a is upregulated during I/R in rat myocardium [29]. In the current study, miR146a-5p represented a positive correlation with insulin resistance and levels of insulin and FBS. Transfection of adipocytes with a miR146a inhibitor resulted in reduced insulin sensitivity [52] and another study has shown that patients with diabetes mellitus have lower levels of miR146a in their peripheral blood mononuclear cells compared to controls [53]. The relation of miR146a with insulin and glucose metabolism indicators in the present study, suggests that it might be a response to insulin resistance that is not effective in reducing insulin sensitivity. Furthermore, we found a positive correlation between miR146a-5p with ICAM-1 and VCAM-1, and previous study has shown a relation of this miR with inflammation [54]. Treatment of endothelial cells with LPS and pro-inflammatory cytokines upregulate miR146a and ICAM-1 and VCAM-1 and the present study showed a positive relation between miR146a and these factors [31, 55]. While studies have shown that miR146a can downregulate ICAM-1 and VCAM-1 [31, 55] and it suppresses inflammation by targeting TNF receptor-associated factor 6 (TRAF6) [56], our results showed a positive correlation between miR146a and adhesion molecules. This finding suggests that miR146a-5p levels *in vivo* might not be as high as levels of upregulated miR146a-5p *in vitro* to suppress ICAM-1 and VCAM-1 and other mechanisms might be more effective in regulating ICAM-1 and VCAM-1 expression, and elevation of miR146a-5p might result from the inflammatory milieu in OSA patients or from a compensatory response. The exact mechanism underlying this finding is unclear and further studies are needed to investigate it.

In conclusion, the present study is the first report on the change of circulating miR125a, miR126, and miR146a-5p in patients with OSA, and provides *in vivo* evidence for the relationship of these miRs with pathological consequences of OSA like endothelial dysfunction, and could be potential therapeutic targets for vascular dysfunction and inflammation in OSA patients, although more studies are required in this regard.

The present study was conducted on subjects with PSG-confirmed OSA and without diabetes and matched according to age, sex, and BMI. While there are some limitations, the sample size and cross-sectional design limited us to conclude a causal relation, and direct measurement of vascular functions was not taken.

## Supporting information

S1 Data Raw data. (XLS)

## References

[1] Lévy P, Kohler M, McNicholas WT, Barbé F, McEvoy RD, Somers VK, et al. Obstructive sleep apnoea syndrome. Nature Reviews Disease Primers. 2015;1(1):15015. doi: 10.1038/nrdp.2015.15 27188535

[2] Senaratna CV, Perret JL, Lodge CJ, Lowe AJ, Campbell BE, Matheson MC, et al. Prevalence of obstructive sleep apnea in the general population: A systematic review. Sleep Med Rev. 2017;34:70–81. Epub 2016/08/29. doi: 10.1016/j.smrv.2016.07.002 . 27568340

[3] Eisele HJ, Markart P, Schulz R. Obstructive Sleep Apnea, Oxidative Stress, and Cardiovascular Disease: Evidence from Human Studies. Oxid Med Cell Longev. 2015;2015:608438. Epub 2015/07/15. doi: 10.1155/2015/608438 ; PubMed Central PMCID: PMC4475750. 26167241 PMC4475750

[4] Wang N, Khan SA, Prabhakar NR, Nanduri J. Impairment of pancreatic β-cell function by chronic intermittent hypoxia. Exp Physiol. 2013;98(9):1376–85. Epub 2013/05/28. doi: 10.1113/expphysiol.2013.072454 ; PubMed Central PMCID: PMC3756548. 23709585 PMC3756548

[5] Tuleta I, França CN, Wenzel D, Fleischmann B, Nickenig G, Werner N, et al. Intermittent Hypoxia Impairs Endothelial Function in Early Preatherosclerosis. Adv Exp Med Biol. 2015;858:1–7. Epub 2015/05/29. doi: 10.1007/5584_2015_114 . 26017722

[6] Orrù G, Storari M, Scano A, Piras V, Taibi R, Viscuso D. Obstructive Sleep Apnea, oxidative stress, inflammation and endothelial dysfunction-An overview of predictive laboratory biomarkers. Eur Rev Med Pharmacol Sci. 2020;24(12):6939–48. Epub 2020/07/08. doi: 10.26355/eurrev_202006_21685 . 32633387

[7] Lavie L. Oxidative stress inflammation and endothelial dysfunction in obstructive sleep apnea. Front Biosci (Elite Ed). 2012;4(4):1391–403. Epub 2011/12/29. doi: 10.2741/469 . 22201964

[8] Wang J, Yu W, Gao M, Zhang F, Gu C, Yu Y, et al. Impact of Obstructive Sleep Apnea Syndrome on Endothelial Function, Arterial Stiffening, and Serum Inflammatory Markers: An Updated Meta-analysis and Metaregression of 18 Studies. J Am Heart Assoc. 2015;4(11):e002454. doi: 10.1161/JAHA.115.002454 . 26567373 PMC4845236

[9] Badran M, Golbidi S, Ayas N, Laher I. Nitric Oxide Bioavailability in Obstructive Sleep Apnea: Interplay of Asymmetric Dimethylarginine and Free Radicals. Sleep Disord. 2015;2015:387801–. Epub 2015/05/06. doi: 10.1155/2015/387801 . 26064689 PMC4438195

[10] O’Brien J, Hayder H, Zayed Y, Peng C. Overview of MicroRNA Biogenesis, Mechanisms of Actions, and Circulation. Front Endocrinol (Lausanne). 2018;9:402. Epub 2018/08/21. doi: 10.3389/fendo.2018.00402 ; PubMed Central PMCID: PMC6085463. 30123182 PMC6085463

[11] Sánchez-de-la-Torre M, Khalyfa A, Sánchez-de-la-Torre A, Martinez-Alonso M, Martinez-García M, Barceló A, et al. Precision Medicine in Patients With Resistant Hypertension and Obstructive Sleep Apnea: Blood Pressure Response to Continuous Positive Airway Pressure Treatment. J Am Coll Cardiol. 2015;66(9):1023–32. Epub 2015/09/01. doi: 10.1016/j.jacc.2015.06.1315 . 26314530

[12] Li K, Wei P, Qin Y, Wei Y. MicroRNA expression profiling and bioinformatics analysis of dysregulated microRNAs in obstructive sleep apnea patients. Medicine (Baltimore). 2017;96(34):e7917. Epub 2017/08/24. doi: 10.1097/MD.0000000000007917 ; PubMed Central PMCID: PMC5572039. 28834917 PMC5572039

[13] Santamaria-Martos F, Benítez I, Ortega F, Zapater A, Giron C, Pinilla L, et al. Circulating microRNA profile as a potential biomarker for obstructive sleep apnea diagnosis. Sci Rep. 2019;9(1):13456–. doi: 10.1038/s41598-019-49940-1 . 31530881 PMC6748919

[14] Khalyfa A, Zhang C, Khalyfa AA, Foster GE, Beaudin AE, Andrade J, et al. Effect on Intermittent Hypoxia on Plasma Exosomal Micro RNA Signature and Endothelial Function in Healthy Adults. Sleep. 2016;39(12):2077–90. doi: 10.5665/sleep.6302 27634792 PMC5103796

[15] Wang Y, Tan J, Wang L, Pei G, Cheng H, Zhang Q, et al. MiR-125 Family in Cardiovascular and Cerebrovascular Diseases. Frontiers in cell and developmental biology. 2021;9:799049. Epub 2021/12/21. doi: 10.3389/fcell.2021.799049 ; PubMed Central PMCID: PMC8674784. 34926475 PMC8674784

[16] Hijmans JG, Levy Ma, Garcia V, Lincenberg GM, Diehl KJ, Greiner JJ, et al. Insufficient sleep is associated with a pro-atherogenic circulating microRNA signature. Experimental Physiology. 2019;104(6):975–82. doi: 10.1113/EP087469 31016755 PMC6544492

[17] Díaz I, Calderón-Sánchez E, Toro RD, Ávila-Médina J, de Rojas-de Pedro ES, Domínguez-Rodríguez A, et al. miR-125a, miR-139 and miR-324 contribute to Urocortin protection against myocardial ischemia-reperfusion injury. Scientific reports. 2017;7(1):8898. Epub 2017/08/23. doi: 10.1038/s41598-017-09198-x ; PubMed Central PMCID: PMC5566224. 28827743 PMC5566224

[18] Wang J, Wu Q, Yu J, Cao X, Xu Z. miR-125a-5p inhibits the expression of NLRP3 by targeting CCL4 in human vascular smooth muscle cells treated with ox-LDL. Exp Ther Med. 2019;18(3):1645–52. Epub 2019/07/01. doi: 10.3892/etm.2019.7717 . 31410121 PMC6676174

[19] Wang S, Aurora AB, Johnson BA, Qi X, McAnally J, Hill JA, et al. The Endothelial-Specific MicroRNA miR-126 Governs Vascular Integrity and Angiogenesis. Developmental Cell. 2008;15(2):261–71. doi: 10.1016/j.devcel.2008.07.002 18694565 PMC2685763

[20] Schober A, Nazari-Jahantigh M, Wei Y, Bidzhekov K, Gremse F, Grommes J, et al. MicroRNA-126-5p promotes endothelial proliferation and limits atherosclerosis by suppressing Dlk1. Nature medicine. 2014;20(4):368–76. doi: 10.1038/nm.3487 24584117 PMC4398028

[21] Zhou J, Li Y-S, Nguyen P, Wang K-C, Weiss A, Kuo Y-C, et al. Regulation of Vascular Smooth Muscle Cell Turnover by Endothelial Cell–Secreted MicroRNA-126. 2013;113(1):40–51. doi: 10.1161/CIRCRESAHA.113.280883 PMC3772783 23603512

[22] Alique M, Bodega G, Giannarelli C, Carracedo J, Ramírez R. MicroRNA-126 regulates Hypoxia-Inducible Factor-1α which inhibited migration, proliferation, and angiogenesis in replicative endothelial senescence. Sci Rep. 2019;9(1):7381. doi: 10.1038/s41598-019-43689-3 31089163 PMC6517399

[23] Rippe C, Blimline M, Magerko KA, Lawson BR, LaRocca TJ, Donato AJ, et al. MicroRNA changes in human arterial endothelial cells with senescence: Relation to apoptosis, eNOS and inflammation. Experimental Gerontology. 2012;47(1):45–51. doi: 10.1016/j.exger.2011.10.004 22037549 PMC3245334

[24] Yang X, Niu X, Xiao Y, Lin K, Chen X. MiRNA expression profiles in healthy OSAHS and OSAHS with arterial hypertension: potential diagnostic and early warning markers. Respiratory Research. 2018;19(1):194. doi: 10.1186/s12931-018-0894-9 30285853 PMC6167890

[25] He L, Liao X, Zhu G, Kuang J. miR-126a-3p targets HIF-1α and alleviates obstructive sleep apnea syndrome with hypertension. Human cell. 2020;33(4):1036–45. Epub 2020/08/12. doi: 10.1007/s13577-020-00404-z . 32779153

[26] Gao W, Hua J, Jia Z, Ding J, Han Z, Dong Y, et al. Expression of miR‑146a‑5p in breast cancer and its role in proliferation of breast cancer cells. Oncol Lett. 2018;15(6):9884–8. doi: 10.3892/ol.2018.8589 29928360 PMC6004703

[27] Xu B, Huang Y, Niu X, Tao T, Jiang L, Tong N, et al. Hsa-miR-146a-5p modulates androgen-independent prostate cancer cells apoptosis by targeting ROCK1. 2015;75(16):1896–903. doi: 10.1002/pros.23068 26306811

[28] Shomali N, Mansoori B, Mohammadi A, Shirafkan N, Ghasabi M, Baradaran B. MiR-146a functions as a small silent player in gastric cancer. Biomedicine & Pharmacotherapy. 2017;96:238–45. doi: 10.1016/j.biopha.2017.09.138 28987948

[29] Shu L, Zhang W, Huang G, Huang C, Zhu X, Su G, et al. Troxerutin attenuates myocardial cell apoptosis following myocardial ischemia-reperfusion injury through inhibition of miR-146a-5p expression. J Cell Physiol. 2019;234(6):9274–82. Epub 2018/11/13. doi: 10.1002/jcp.27607 . 30417352

[30] Lin G, Huang J, Chen Q, Chen L, Feng D, Zhang S, et al. miR-146a-5p Mediates Intermittent Hypoxia-Induced Injury in H9c2 Cells by Targeting XIAP. Oxidative Medicine and Cellular Longevity. 2019;2019:6581217. Epub 2019/06/18. doi: 10.1155/2019/6581217 ; PubMed Central PMCID: PMC6530234. 31205587 PMC6530234

[31] Cheng HS, Sivachandran N, Lau A, Boudreau E, Zhao JL, Baltimore D, et al. MicroRNA-146 represses endothelial activation by inhibiting pro-inflammatory pathways. EMBO Mol Med. 2013;5(7):1017–34. Epub 2013/06/05. doi: 10.1002/emmm.201202318 ; PubMed Central PMCID: PMC3721471. 23733368 PMC3721471

[32] Mohammadi H, Rezaei M, Sharafkhaneh A, Khazaie H, Ghadami MR. Serum testosterone/cortisol ratio in people with obstructive sleep apnea. 2020;34(1):e23011. doi: 10.1002/jcla.23011 31549459 PMC6977109

[33] Khazaie H, Negahban S, Ghadami MR, Sadeghi Bahmani D, Holsboer-Trachsler E, Brand S. Among middle-aged adults, snoring predicted hypertension independently of sleep apnoea. Journal of International Medical Research. 2018;46(3):1187–96. doi: 10.1177/0300060517738426 29322844 PMC5972254

[34] Schmittgen TD, Livak KJ. Analyzing real-time PCR data by the comparative CT method. Nature Protocols. 2008;3(6):1101–8. doi: 10.1038/nprot.2008.73 18546601

[35] Hao L, Wang XG, Cheng JD, You SZ, Ma SH, Zhong X, et al. The up-regulation of endothelin-1 and down-regulation of miRNA-125a-5p, -155, and -199a/b-3p in human atherosclerotic coronary artery. Cardiovasc Pathol. 2014;23(4):217–23. Epub 2014/06/01. doi: 10.1016/j.carpath.2014.03.009 . 24877885

[36] Paterson MR, Kriegel AJ. MiR-146a/b: a family with shared seeds and different roots. Physiol Genomics. 2017;49(4):243–52. Epub 2017/02/19. doi: 10.1152/physiolgenomics.00133.2016 ; PubMed Central PMCID: PMC5407182. 28213571 PMC5407182

[37] Ma S, Tian XY, Zhang Y, Mu C, Shen H, Bismuth J, et al. E-selectin-targeting delivery of microRNAs by microparticles ameliorates endothelial inflammation and atherosclerosis. Sci Rep. 2016;6:22910. Epub 2016/03/10. doi: 10.1038/srep22910 ; PubMed Central PMCID: PMC4783714. 26956647 PMC4783714

[38] Wang J, Yan F, Zhao Q, Zhan F, Wang R, Wang L, et al. Circulating exosomal miR-125a-3p as a novel biomarker for early-stage colon cancer. Scientific reports. 2017;7(1):4150. doi: 10.1038/s41598-017-04386-1 28646161 PMC5482839

[39] Wang Y, Zeng G, Jiang Y. The Emerging Roles of miR-125b in Cancers. Cancer Manag Res. 2020;12:1079–88. doi: 10.2147/CMAR.S232388 . 32104088 PMC7024862

[40] Ke H, Zhang X, Cheng L, Fan Y, Xiao S, Ma Y, et al. Bioinformatic analysis to explore key genes associated with brain ischemia-reperfusion injury in rats. Int J Neurosci. 2019;129(10):945–54. Epub 2019/03/20. doi: 10.1080/00207454.2019.1595615 . 30889366

[41] Zhaolin Z, Jiaojiao C, Peng W, Yami L, Tingting Z, Jun T, et al. OxLDL induces vascular endothelial cell pyroptosis through miR-125a-5p/TET2 pathway. J Cell Physiol. 2019;234(5):7475–91. Epub 2018/10/30. doi: 10.1002/jcp.27509 . 30370524

[42] Pan Q, Liao X, Liu H, Wang Y, Chen Y, Zhao B, et al. MicroRNA-125a-5p alleviates the deleterious effects of ox-LDL on multiple functions of human brain microvessel endothelial cells. Am J Physiol Cell Physiol. 2017;312(2):C119–c30. Epub 2016/12/03. doi: 10.1152/ajpcell.00296.2016 . 27903586

[43] Banerjee S, Cui H, Xie N, Tan Z, Yang S, Icyuz M, et al. miR-125a-5p regulates differential activation of macrophages and inflammation. The Journal of biological chemistry. 2013;288(49):35428–36. Epub 2013/10/24. doi: 10.1074/jbc.M112.426866 ; PubMed Central PMCID: PMC3853290. 24151079 PMC3853290

[44] Wade SM, Ohnesorge N, McLoughlin H, Biniecka M, Carter SP, Trenkman M, et al. Dysregulated miR-125a promotes angiogenesis through enhanced glycolysis. EBioMedicine. 2019;47:402–13. Epub 2019/08/31. doi: 10.1016/j.ebiom.2019.08.043 ; PubMed Central PMCID: PMC6796559. 31466915 PMC6796559

[45] Huang H, Huang J, Yao J, Li N, Yang Z. miR-125a regulates HAS1 and inhibits the proliferation, invasion and metastasis by targeting STAT3 in non-small cell lung cancer cells. Journal of cellular biochemistry. 2020;121(5–6):3197–207. Epub 2020/01/14. doi: 10.1002/jcb.29586 . 31930562

[46] Meszaros M, Kis A, Kunos L, Tarnoki AD, Tarnoki DL, Lazar Z, et al. The role of hyaluronic acid and hyaluronidase-1 in obstructive sleep apnoea. Scientific reports. 2020;10(1):19484. Epub 2020/11/12. doi: 10.1038/s41598-020-74769-4 ; PubMed Central PMCID: PMC7655850. 33173090 PMC7655850

[47] Ye P, Liu J, He F, Xu W, Yao K. Hypoxia-induced deregulation of miR-126 and its regulative effect on VEGF and MMP-9 expression. Int J Med Sci. 2013;11(1):17–23. doi: 10.7150/ijms.7329 . 24396282 PMC3880987

[48] Zampetaki A, Kiechl S, Drozdov I, Willeit P, Mayr U, Prokopi M, et al. Plasma microRNA profiling reveals loss of endothelial miR-126 and other microRNAs in type 2 diabetes. Circ Res. 2010;107(6):810–7. Epub 2010/07/24. doi: 10.1161/CIRCRESAHA.110.226357 . 20651284

[49] Harris TA, Yamakuchi M, Ferlito M, Mendell JT, Lowenstein CJ. MicroRNA-126 regulates endothelial expression of vascular cell adhesion molecule 1. Proceedings of the National Academy of Sciences. 2008;105(5):1516–21. doi: 10.1073/pnas.0707493105 18227515 PMC2234176

[50] Fu X, Niu T, Li X. MicroRNA-126-3p Attenuates Intracerebral Hemorrhage-Induced Blood-Brain Barrier Disruption by Regulating VCAM-1 Expression. Frontiers in Neuroscience. 2019;13. doi: 10.3389/fnins.2019.00866 31474826 PMC6707088

[51] Tang F, Yang TL. MicroRNA-126 alleviates endothelial cells injury in atherosclerosis by restoring autophagic flux via inhibiting of PI3K/Akt/mTOR pathway. Biochem Biophys Res Commun. 2018;495(1):1482–9. Epub 2017/12/06. doi: 10.1016/j.bbrc.2017.12.001 . 29203244

[52] Roos J, Dahlhaus M, Funcke J-B, Kustermann M, Strauss G, Halbgebauer D, et al. miR-146a regulates insulin sensitivity via NPR3. Cell Mol Life Sci. 2021;78(6):2987–3003. Epub 2020/11/18. doi: 10.1007/s00018-020-03699-1 . 33206203 PMC8004521

[53] Yang M, Ye L, Wang B, Gao J, Liu R, Hong J, et al. Decreased miR-146 expression in peripheral blood mononuclear cells is correlated with ongoing islet autoimmunity in type 1 diabetes patients 1miR-146. J Diabetes. 2015;7(2):158–65. Epub 2014/05/07. doi: 10.1111/1753-0407.12163 . 24796653

[54] Prada I, Gabrielli M, Turola E, Iorio A, D’Arrigo G, Parolisi R, et al. Glia-to-neuron transfer of miRNAs via extracellular vesicles: a new mechanism underlying inflammation-induced synaptic alterations. Acta Neuropathol. 2018;135(4):529–50. Epub 2018/01/06. doi: 10.1007/s00401-017-1803-x ; PubMed Central PMCID: PMC5978931. 29302779 PMC5978931

[55] Feng LL, Xin WN, Tian XL. MALAT1 modulates miR-146’s protection of microvascular endothelial cells against LPS-induced NF-κB activation and inflammatory injury. Innate Immun. 2019;25(7):433–43. Epub 2019/07/12. doi: 10.1177/1753425919861427 ; PubMed Central PMCID: PMC6900645. 31291804 PMC6900645

[56] Yan F, Wufuer D, Ding J, Wang J. MicroRNA miR-146a-5p inhibits the inflammatory response and injury of airway epithelial cells via targeting TNF receptor-associated factor 6. Bioengineered. 2021;12(1):1916–26. Epub 2021/05/19. doi: 10.1080/21655979.2021.1927545 ; PubMed Central PMCID: PMC8806598. 34002665 PMC8806598

